# Metformin Versus Insulin for Gestational Diabetes: Cognitive and Neuropsychological Profiles of Children Aged 9 years

**DOI:** 10.1097/DBP.0000000000001233

**Published:** 2023-11-28

**Authors:** Elisa Paavilainen, Anna Nyman, Harri Niinikoski, Hilkka Nikkinen, Riitta Veijola, Marja Vääräsmäki, Päivi Tossavainen, Tapani Rönnemaa, Kristiina Tertti

**Affiliations:** * Department of Pediatrics and Adolescent Medicine, University of Turku and Turku University Hospital, Turku, Finland;; † Department of Psychology and Speech-Language Pathology, University of Turku, Turku, Finland;; ‡ Department of Obstetrics and Gynecology, Research Unit of Clinical Medicine, Medical Research Center, University of Oulu and Oulu University Hospital, Oulu, Finland;; § Department of Pediatrics, Research Unit of Clinical Medicine, Medical Research Center, University of Oulu and Oulu University Hospital, Oulu, Finland;; ║ Department of Medicine, University of Turku and Division of Medicine, Turku University Hospital, Turku, Finland; and; ¶ Department of Obstetrics and Gynecology, University of Turku and Turku University Hospital, Turku, Finland.

**Keywords:** gestational diabetes mellitus, metformin, offspring, cognitive development, neuropsychological functions

## Abstract

**Objective:**

We compared cognitive profile and neuropsychological performance in 9-year-old offspring of mothers who were treated with metformin or insulin for gestational diabetes mellitus (GDM).

**Methods:**

A total of 172 children whose mothers were randomly assigned to receive either metformin or insulin for GDM were studied at the age of 9 years. Of these children, 127 were from Turku, Finland (63 metformin and 64 insulin), and 45 from Oulu, Finland (19 metformin and 26 insulin). Clinical and demographic background characteristics were obtained at enrolment, birth, and 9-year follow-up. Cognitive profiles were examined at age 9 years with the Wechsler Intelligence Scale for Children. Neuropsychological functions were examined with 2 subtests of the Developmental Neuropsychological Assessment test battery assessing comprehension of instructions and narrative memory, Trail Making Test assessing attention and with Behavioral Rating Inventory of Executive Functioning, including parent-rated and teacher-rated evaluations. Academic functioning was studied with reading fluency subtest of the Screening test for reading, writing, and calculus for first to sixth grades and information about educational support received at school reported by parents.

**Results:**

The cognitive profiles, including indexes of verbal comprehension, perceptual reasoning, working memory, and processing speed, did not differ significantly between metformin-treated and insulin-treated groups. Significant differences were not found between the treatment groups in assessed neuropsychological functions, reading fluency, or received level of support at school.

**Conclusion:**

Cognitive and neuropsychological outcomes were similar in 9-year-old children whose mothers had either metformin or insulin treatment of GDM.

## Abstract 2

This article has supplementary material on the web site: www.jdbp.org.

Gestational diabetes mellitus (GDM) is defined as a condition in which hyperglycemia develops and is diagnosed for the first time during pregnancy.^1^ Intrauterine exposure to hyperglycemia, hyperinsulinemia, and proinflammatory mediators in GDM can affect the long-term neurodevelopment of children.^2^ However, these findings have been contradictory,^3–6^ and the study designs have been heterogeneous.^7,8^ Two recent studies report that particularly GDM combined with maternal pre-pregnancy overweight or obesity may lead to transgenerational brain changes^9^ or to weaker neurodevelopmental skills in offspring, although being still within the mean normative range in this population.^10^

Metformin is increasingly used in the treatment of GDM. However, metformin crosses the placenta with fetal levels similar to maternal concentrations.^11^ It has also shown to cross the blood-brain barrier in experimental animals and in humans and to exert various neurophysiological actions.^12^

Mice studies have shown that male and female offspring may develop different metabolic phenotypes after similar exposure, e.g., GDM or metformin medication during pregnancy.^13,14^ In our previous studies, boys in the metformin group had better high-density lipoprotein cholesterol^15^ and adiponectin^16^ concentration than the boys in the insulin group at the age of 9 years. Still, in short term, metformin has been considered as a safe option for both the mother and the child.^17–20^ Thus, possible long-term effects on neurocognitive development of the offspring after prenatal exposure of metformin treatment of GDM are important to evaluate.

Previously, neurodevelopment of offspring in randomized studies comparing maternal metformin or insulin treatment of GDM has been followed only until the age of 18 months^21^ or 2 years,^22,23^ and in 1 population-based cohort study, the questionnaire of offspring psychosocial and behavioral indices was gathered before entering school.^24^ In these 4 studies, no significant differences were found between the 2 treatment groups.

The aim of this study was to assess possible long-term effects of prenatal metformin exposure on cognitive and neuropsychological performance in 9-year-old children. The study subjects were children born to mothers with GDM randomized to metformin or insulin treatment. Cognitive and neuropsychological outcomes between children of metformin and insulin groups were also analyzed in subgroups by sex. The age of 9 years, just before the onset of puberty and after completing the second grade of primary school education, was considered the most appropriate age to compare the cognitive and neuropsychological variables between the offspring of the 2 treatment groups.

## METHODS

### Study Subjects

**Figure 1.**
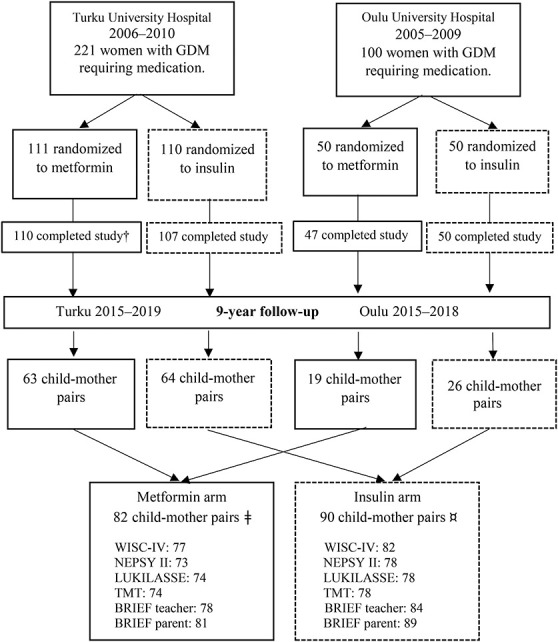
Participants of the 2 original randomized controlled trials and those of the 9-year follow-up study. †Of the 110 participants, who completed the original study in the metformin group in Turku, 3 offspring were excluded because 1 child had valproate syndrome, 1 child had down syndrome, and 1 child was stillborn. In the 9-year follow-up of 82 participants in the metformin group, 2 children were excluded (Swedish language), and for 2 children, a psychologist was not available for testing. ¤Of the 90 participants in the insulin group, 7 children were excluded because 1 child had attended a psychological test within less than a year, 8 children had Swedish as a school language, and for 1 child, a psychologist was not available for testing. BRIEF, Behavioral Rating Inventory of Executive Functioning; GDM, gestational diabetes mellitus; LUKILASSE, Screening test for reading, writing, and calculus for first to sixth grades; NEPSY II, developmental neuropsychological assessment test; TMT, Trail Making Test; WISC, Wechsler Intelligence Scale for Children.

This was a prospective follow-up study in the offspring of 2 previously published Finnish randomized controlled trials with similar study designs,^25,26^ comparing metformin and insulin treatment of GDM. The study design of this follow-up study has been previously described in detail.^15^ A total of 172 children participated in a 9-year follow-up study, comprising 55% of all eligible children (n = 311) from the 2 original trials (Fig. 1). For neuropsychological assessment, 159 of 311 children (51%) participated. In total, 82 of the participating children (48%) were born to mothers who were randomly assigned at 17 to 34 gestational weeks to treatment with metformin and 90 (52%) were born to mothers assigned to insulin treatment. Furthermore, in the metformin group, 27% of the mothers (22 of 82) received additional insulin to achieve sufficient glucose balance. The aim of this study was to investigate the safety of metformin; thus, children born to mothers originally randomized to receive metformin were handled as 1 group in all analyses, including those whose mothers needed additional insulin.

This 9-year follow-up study was conducted at 2 sites, Turku University Hospital in Southwest Finland and Oulu University Hospital in Northern Finland, between 2015 and 2019. Examination included measurements of growth, body composition, and blood tests for metabolism, and these results were reported previously.^15^ Study examinations of the children were arranged during 1 day in the following order: fasting blood samples, oral glucose tolerance test, anthropometric measurements,^15^ and after lunch, cognitive and neuropsychological assessments and radiological imaging studies assessing adiposity.^16^ Furthermore, anthropometric data (height and weight) of the mothers were gathered during the children's study visit. Parental demographic and lifestyle data together with paternal anthropometric values and children's school-related factors were collected from the parents before the study visits using questionnaires designed for the purposes of the present study. In addition, teachers and parents filled in questionnaires about their child's executive functioning before the study visit. In Finland, basic education starts the year children turn 7 years, and at the age of 9 years, they are completing their second or started their third grade at school. Nine children, 2 from the metformin group and 7 from the insulin group, were excluded from the neuropsychological assessments: 1 child had attended a similar psychological test within less than a year and 8 children did not have Finnish as a school language. Furthermore, the neuropsychological assessment was not completed for 3 children because of scheduling reasons, and 1 child was not able to attend the assessment because of nausea and feeling ill.

Written informed consent was obtained from each mother, child, and father. The assessors were blinded to the treatment allocation of the mothers. The 9-year follow-up study was registered with the Clinical Trials Registry (NCT02417090) and approved by the Ethics Committee of the Hospital District of Southwest Finland (ETMK 31/2015, April 27, 2015).

### Cognitive and Neuropsychological Assessments

Neuropsychological test battery for the purposes of this study was designed to cover essential functions of development and school performance of 9-year-old children. Neuropsychological assessments were performed in Finnish. Two psychologists and 3 final-stage psychology students under the guidance of an experienced psychologist made the assessments over a 4-year period.

### Cognitive Development

*Cognitive development* of the children was assessed using the Finnish translation of Wechsler Intelligence Scale for Children, Fourth Edition (WISC-IV).^27,28^ Full-Scale Intelligence Quotient (FSIQ) was used as a measure of general intelligence. FSIQ comprises 4 indexes derived from 10 subtests. The Verbal Comprehension Index measures the ability of verbal reasoning and acquired knowledge, Perceptual Reasoning Index (PRI) measures perceptual organization and logical reasoning, Working Memory Index measures working memory and attention, and Processing Speed Index measures the speed of mental and fine motor processing. WISC-IV indexes were calculated according to age-appropriate Finnish norms (mean 100, SD 15)^28^ and used as a continuous variable. Based on clinical significance, the cutoff was set to <85 points (−1 SD) in WISC-IV indexes to identify children whose results were at least slightly below normal.^27,28^

### Neuropsychological Performance

*Language functions* were assessed using a Developmental Neuropsychological Assessment (NEPSY II)^29,30^ subtest called Comprehension of Instructions, assessing the ability to receive and process oral instructions of increasing complexity.

*Memory functions* were assessed using a NEPSY II^29,30^ subtest called Narrative Memory, assessing memory for logical verbal story under free and cued recall. Scores of the NEPSY II measures were based on age-appropriate Finnish norms (mean 10, SD 3)^29,30^ and used as a continuous variable. The cutoff was set to <8 standard scores (−1 SD) to identify results that were at least slightly below normal.^29,30^

*Attention regulation* was assessed with the Trail Making Test (TMT) for children,^31^ consisting of 2 parts: TMT A, in which the respondent is asked to connect randomly arranged circles containing numbers and requires visual tracking and simple set-sifting, and TMT B, in which the respondent has to alternate between numbers and letters and requires visual tracking and complex set-sifting. The time in minutes needed to complete each part as quickly as possible was used as a measure of performance and used as a continuous variable.

*Executive functions* in daily life were assessed using both teacher and parent forms of the Finnish translation of the Behavior Rating Inventory of Executive Function (BRIEF).^32^ BRIEF forms consist of 86 items with a 3-point Likert scale, and these items consist of 8 subscales that form 2 indexes. The Behavioral Regulation Index is a composite score of Inhibit (ability to resist impulse), Shift (making transitions between tasks and mindsets), and Emotional Control (regulation of emotional responses). The Metacognition Index is a composite score of Initiate (starting an activity independently), Working Memory (holding information to complete a task), Plan/Organize (planning and organizing ahead for future events), Organization of Materials (sorting and organizing things), and Monitor (assessing one's own performance for proper goal attainment). The Global Executive Composite Score combines Behavioral Regulation Index and Metacognition Index. The age-specific and sex-specific standardized T-scores on the subscales and index scores were used to measure outcomes^32^ and used as a continuous variable. Pre-established cutoff T-score > 64 was used to indicate clinically significant symptoms.^32^ Only consistently filled-in questionnaires were used in the analyses.^32^

### Academic Functioning

*Reading fluency* was assessed with a subtest of the Screening test for reading, writing, and calculus for first to sixth grades (LUKILASSE). In that test, the study participants read as many words as possible from the word list in 2 minutes and correctly read words are counted.^33^ Standard scores at or below −1.34 SD were considered as clearly below grade level.^33^

*School-related factors*—information about the level of educational support—were collected from parents. Educational support is divided into 3 levels, i.e., general, intensified, and special support. All students are covered by general support. Intensified support means a part-time special education in a specific area, such as literacy or mathematics, and special support means full-time special education that is intended for children with a long-term need of support and includes mainly individualized education plans in one or several subjects.

### Data Analysis

Owing to a relatively small number of children in both study groups, post hoc power analysis was performed to evaluate the reliability of the results. Assuming at least noninferiority between the study groups, the required sample sizes were calculated to attain 80% statistical power on 95% significance level with 10 points as a noninferiority margin using the observed group means and pooled SD. We chose a 10-point difference because we were interested in clinically significant difference that might affect the performance of the children. In this setup, we found that sufficient total sample size to assess noninferiority for FSIQ is n = 95 subjects and for PRI n = 216 subjects. This analysis was performed using R: a language and environment for statistical computing, version 4.2.3 (R Foundation for Statistical Computing, Vienna, Austria) and epiR package, version 2.0.57 (Stevenson et al.).^34^

The Kolmogorov-Smirnov test was used to analyze whether the variables were normally distributed, and the Shapiro-Wilk test was used to test the normality of the subgroups of boys and girls (n < 50). Continuous variables are described using means, SDs or medians, and interquartile ranges (IQR). Categorical variables are described using frequencies and percentages. Between-group comparisons in continuous variables were performed using Student's *t*test for normally distributed data and the Mann-Whitney *U* test for skewed data. The chi-square test or Fisher's exact test was used for categorical variables. Potential differences in boys and girls between the treatment groups were explored using subgroup analysis. Multivariable regression analysis was used to adjust the results for parental education level and sex. The IBM SPSS Statistics (IBM Corp, Armonk, NY) software package, version 27.0, was used, and a *p* value of <0.05 was set to indicate statistical significance.

## RESULTS

### Study Subjects

A total of 172 children (82 in the metformin group and 90 in the insulin group) were followed up at 9 years. The means or medians of cognitive development, neuropsychological performance, and academic functioning were similar in offspring assessed in the 2 study sites (Turku n = 127, Oulu n = 45), which allowed further analysis to be made as a single group. The results of neuropsychological assessments were eligible in 159 participants, which is 51% of the 311 children in the original cohort and 77 (48%) of these belonged to the metformin group and 82 (52%) belonged to the insulin group (Fig. 1). The BRIEF questionnaires of 13 children who were excluded or whose test results were not obtained were received and included in the analyses (Fig. 1). The children participated in the neuropsychological assessment the year they turned 9 years. The median age of the participants was 9.1 years (range 8.8–9.5) in the metformin group and 9.0 years (range 8.8–9.7) in the insulin group (Table 1). Fifteen percent of the neuropsychological assessments (24 of 159: 10 [13%] in the metformin group and 14 [17.1%] in the insulin group) were conducted on a day separate from other study examinations. The means or medians of neuropsychological assessments were similar despite the difference in survey dates.

**Table 1.** Pregnancy, Neonatal, and 9-Yr Follow-Up Characteristics of the Participants in the 9-Yr Follow-Up Study

	Metformin	Insulin	*p*
Mother, pregnancy	n = 77	n = 82	
Age (yr), 1st antenatal visit	32.7 ± 4.8	32.5 ± 5.3	0.72
Ethnicity, White, n (%)	76 (98.7)	82 (100)	0.30
BMI (kg/m^2^), 1st antenatal visit	29.0 (25.0–33.0)	28.0 (25.7–33.0)	0.87ǂ
Total weight gain during pregnancy (kg)	8.3 ± 4.7	8.6 ± 5.4	0.71
HbA1c prior randomization (%)	5.5 ± 0.4	5.6 ± 0.4	0.39
HbA1c at 36 gestational weeks (%)	5.6 ± 0.3	5.7 ± 0.4	0.42
Gestational weeks at randomization (wk)	30.4 (29.2–32.0)	30.8 (29.3–32.0)	0.43ǂ
Duration of insulin/metformin medication (wk)	8.6 (7.1–10.6)	8.4 (6.7–10.4)	0.52ǂ
Gestational weeks at birth (wk)	39.0 (38.4–40.1)	39.1 (38.4–40.3)	0.66ǂ
Prematurity (delivery <37 gestational weeks)	6 (7.8)	3 (3.7)	0.26†
Child, newborn	n = 77	n = 82	
Birth weight (g)	3620 ± 490	3571 ± 542	0.56
Birth weight (SD)	0.18 ± 1.08	0.07 ± 1.17	0.55
Birth weight < −2 SD	3 (3.9)	1 (1.2)	0.28†
Apgar points at 1 min	9.0 (9.0–9.0)	9.0 (8.0–9.0)	0.22ǂ
Apgar points at 5 min	9.0 (9.0–9.0)	9.0 (9.0–9.0)	0.87ǂ
Apgar points at 15 min	9.0 (9.0–10.0)	9.0 (9.0–10.0)	0.56ǂ
Umbilical artery pH	7.28 ± 0.09	7.28 ± 0.08	0.997
Hypoglycemia, need for IV glucose	16 (20.8)	14 (17.1)	0.55
Child, 9 yr	n = 77	n = 82	
Age	9.1 (9.0–9.1)	9.0 (9.0–9.1)	
Boys/girls	39 (50.6)/38 (49.4)	38 (46.3)/44 (53.7)	0.64
BMI (kg/m^2^)	17.45 (16.3–19.4)	17.85 (16.1–20.7)	0.63ǂ
Overweight or obese (adjusted BMI ≥25.0)¤	20 (26.3)	30 (36.6)	0.17
Waist:height ratio	0.43 (0.41–0.47)	0.44 (0.43–0.49)	0.13
Waist:height ratio >0.5	11 (14.5)	14 (17.1)	0.67
Grade at school, 2nd/3rd	37 (48.1)/40 (51.9)	46 (56.1)/36 (43.9)	0.31
Mother at 9-yr visit	n = 77	n = 82	
Age (yr)	42.3 ± 4.8	41.9 ± 5.4	0.63
BMI (kg/m^2^)	31.1 ± 5.8	31.0 ± 5.3	0.91
Regular smoking	10 (13.0)	12 (14.6)	0.76
Education level			
Lower or upper secondary	37 (52.1)	43 (55.1)	0.71
Postsecondary or higher	34 (47.9)	35 (44.9)	
Father at 9-yr visit	n = 72	n = 75	
Age (yr)	42.0 (39.8–46.3)	42.5 (40.0–48.8)	0.47ǂ
BMI (kg/m^2^)	27.0 (24.7–30.8)	27.8 (24.7–30.5)	0.70ǂ
Regular smoking	18 (25.0)	16 (21.3)	0.60
Education level			
Lower or upper secondary	42 (60.0)	49 (65.3)	0.51
Postsecondary or higher	28 (40.0)	26 (34.7)	

Psychological assessments were performed in 159 participants. Comparison between the groups treated with metformin or insulin for GDM.

Data are expressed as mean ± SD, median (IQR), or n (%). The *t* test or Mann-Whitney *U* test (ǂ) was used for continuous variables, and the χ^2^ or Fisher's exact test (†) was used for categorical variables. Age-specific and sex-specific adjusted BMI cutoff points are used according to Cole et al.^35^ (¤).

BMI, body mass index; GDM, gestational diabetes mellitus; IQR, interquartile ranges.

No significant differences between the participants and nonparticipants of the 9-year follow-up study were found in maternal baseline characteristics of pregnancy, i.e., pre-pregnancy body mass index (BMI), glycemic status, smoking habits, distribution of metformin and insulin treatments, duration of medication, and gestational weeks at delivery. The same was true in neonatal measures, i.e., birth weight, umbilical artery pH, Apgar score, need for IV glucose for hypoglycemia, and sex distribution of the children within the groups (Table S1, Supplemental Digital Content 1, http://links.lww.com/JDBP/A446). Smoking during pregnancy was slightly, but nonsignificantly, different (*p* = 0.060) between the 2 medication groups, and after adjusting for smoking in pregnancy, the results of WISC-IV indexes did not change. No differences were found in the abovementioned baseline characteristics between medication groups of the participants in the 9-year follow-up study (Table S2, Supplemental Digital Content 1, http://links.lww.com/JDBP/A446, Table 1). The characteristics of the 9-year-old children (e.g., sex, BMI, proportion of children with overweight/obesity or waist-height ratio >0.5) were similar in the 2 groups. Furthermore, parental characteristics (e.g., age, BMI, smoking habits and education level) at the 9-year visit were similar between the groups (Table 1).

### Cognitive Development at the Age of 9 Years

The results of Full-Scale Intelligence Quotient, Verbal Comprehension Index, PRI, Working Memory Index, and Processing Speed Index between metformin and insulin groups were similar (Table 2). Adjustment for maternal and paternal educational levels did not change the results. However, when comparing the proportion of children who performed below the average level (<85 standard points) in FSIQ, 28.6% of the children belonged to the metformin group and 16.5% to the insulin group (*p* = 0.070, Table S3, Supplemental Digital Content 1, http://links.lww.com/JDBP/A446). Three children had FISQ < 70 corresponding to severe cognitive impairment (standard points between 50 and 69). Two of these children were boys of the metformin group (FSIQ 64 and 69), and one was a girl of the insulin group (FSIQ 65).

**Table 2.** Cognitive Development, Neuropsychological Performance, and Academic Functioning at the Age of 9 yr

	All Children			Boys			Girls		
	Metformin	Insulin	*p*	Metformin	Insulin	*p*	Metformin	Insulin	*p*
	n = 77	n = 82		n = 39	n = 38		n = 38	n = 44	
Cognitive development^a^									
Full-scale IQ	96.0 ± 15.0	97.8 ± 13.4	0.43	92.3 ± 12.8	93.4 ± 11.0	0.68	99.9 ± 16.2	101.7 ± 14.3	0.60
Verbal comprehension	98.6 ± 13.1	99.9 ± 12.6	0.53	97.1 ± 12.3	96.5 ± 10.8	0.83	100.2 ± 13.9	102.9 ± 13.4	0.38
Perceptual reasoning	96.5 ± 15.0	100.7 ± 15.1	0.081	94.1 ± 14.7	97.3 ± 13.3	0.31	99.0 ± 15.1	103.6 ± 16.1	0.19
Working memory	94.2 ± 12.9	94.6 ± 11.3	0.61ǂ	92.0 ± 12.1	95.0 ± 10.0	0.24	96.5 ± 13.5	94.3 ± 12.3	0.45
Processing speed	100.7 ± 15.2	97.9 ± 14.4	0.23	95.0 ± 11.0	93.6 ± 14.6	0.65	106.7 ± 16.7	101.6 ± 13.2	0.13
Neuropsychological performance^b^									
Comprehension of instructions	10.0 (8.0–12.0)	10.5 (8.8–12.0)	0.26ǂ	8.9 (7.0–11.0)	10.0 (8.0–11.0)	0.34ǂ	11.0 (8.3–12.0)	11.0 (9.0–12.0)	0.61ǂ
Narrative memory	7.1 ± 3.4	7.6 ± 3.9	0.48	6.8 ± 3.3	6.6 ± 3.7	0.77	7.4 ± 3.4	8.3 ± 4.0	0.29
TMT A (s)	23.0 (19.0–30.0)	22.5 (18.0–29.3)	0.55ǂ	23.0 (18.0–28.0)	23.0 (17.0–32.0)	0.99ǂ	23.0 (19.5–30.0)	21.0 (19.0–28.0)	0.41ǂ
TMT B (s)	47.0 (40.0–74.0)	50.0 (38.0–67.3)	0.81ǂ	45.5 (40.0–74.0)	55.0 (39.0–70.0)	0.47ǂ	48.0 (39.5–71.5)	48.0 (38.0–63.0)	0.77ǂ
Academic functioning^c^									
Reading fluency	71.8 ± 19.9	68.4 ± 18.0	0.28	67.4 ± 20.1	66.8 ± 16.1	0.90	76.2 ± 18.9	69.9 ± 19.7	0.16
Educational support									
Intensified, n (%)	3 (3.9)	6 (7.3)		3 (7.7)	4 (10.5)		0 (0)	2 (4.5)	
Special, n (%)	8 (10.4)	1 (1.2)		4 (10.3)	0 (0)		4 (10.5)	1 (2.3)	
Intensified or special, n (%)	11 (14.3)	7 (8.5)	0.063	7 (18.0)	4 (10.5)	0.13	4 (10.5)	3 (6.8)	0.28

Psychological assessments were performed in 159 participants. Comparison between the offspring of the mothers treated with metformin or insulin for GDM.

Data are expressed as mean ± SD, median (IQR) or n (%). The *t* test or Mann-Whitney *U* test (ǂ) was used for continuous variables, and the Fisher's exact test was used for categorical variables.

a Wechsler Intelligence Scale for Children, Fourth Edition (WISC-IV).

b Two subtests of the Developmental Neuropsychological Assessment (NEPSY-II) test battery and TMT.

c One subtest of the Screening test for reading, writing, and calculus for first to sixth grades (LUKILASSE) and information about received support at school.

GDM, gestational diabetes mellitus; IQR, interquartile ranges; TMT, Trail Making Test.

### Neuropsychological and Academic Functions at the Age of 9 Years

The results of the 2 subtests of NEPSY II, TMT A-time and B-time, and reading fluency test by LUKILASSE were similar in the children of the metformin and insulin groups (Table 2). Grade levels at school and proportion of children receiving educational support (Table 2) were similar in the 2 groups. In addition, executive function profiles at school and at home and the proportion of the children reported to have clinically significant symptoms were similar in the 2 study groups (Table 3, Table S4, Supplemental Digital Content 1, http://links.lww.com/JDBP/A446). At the time of the neuropsychological assessment, half of the children were completing second grade and half of the children were completing third grade at school (Table 2). Eleven children (14.3%) of the metformin group and 7 children (8.5%) of the insulin group received intensified or special support at school (*p* = 0.063 between groups; Table 2).

**Table 3.** Executive Functioning in Daily Life at the Age of 9 yr as Assessed by the Teacher and Parent

	All Children		
	Metformin	Insulin	*p*
Executive functioning at school (n = 162)	n = 78	n = 84	
Behavioral regulation index	48.5 (45–60)	48.0 (45–57)	0.55
Clinically significant problems at school, n (%)	11 (14)	7 (8)	0.24
Metacognition index	51.0 (44–60)	50.0 (45–60)	0.68
Clinically significant problems at school, n (%)	13 (17)	14 (17)	0.97
Global executive composite scores	50.0 (45–61)	49.5 (45–60)	0.93
Clinically significant problems at school, n (%)	13 (17)	12 (14)	0.65
Executive functioning at home (n = 170)	n = 81	n = 89	
Behavioral regulation index	43.0 (39–48)	42.0 (38–50)	0.54
Clinically significant problems at home, n (%)	4 (5)	2 (2)	0.34†
Metacognition index	44.0 (39–52)	44.0 (38–48)	0.37
Clinically significant problems at home, n (%)	2 (3)	2 (2)	0.93†
Global executive composite scores	45.0 (38–50)	43.0 (38–48)	0.39
Clinically significant problems at home, n (%)	2 (3)	3 (3)	0.72†

Medians of BRIEF indexes and proportion of children who had clinically significant problems (scores above 64) in BRIEF at school or at home. Comparison between the offspring of the mothers treated with metformin or insulin for GDM.

Data are expressed as median (IQR) or n (%). Scores above 64 are used to indicate clinically significant problems. The Mann-Whitney *U* test was used for continuous variables, and the χ^2^ or Fisher's exact test (†) was used for categorical variables.

BRIEF, Behavior Rating Inventory of Executive Function; GDM, gestational diabetes mellitus; IQR, interquartile ranges.

## DISCUSSION

In this follow-up study, which comprised one hundred fifty-nine 9-year-old children born to mothers with GDM, we found no statistically significant differences in cognitive or neuropsychological outcomes between the offspring of mothers who were treated with either metformin or insulin.

It has been shown that metformin crosses the placenta^11^ and ends up in fetal circulation in similar concentration as in the mother's blood. Furthermore, metformin may also be transported across the blood-brain barrier in the fetal brain like in an adult brain.^12^ Thus, metformin might potentially influence the cognitive development of the offspring of mothers with GDM. Contrarily, metformin has been increasingly studied in adults because of its possible neuroprotective actions in several neurological diseases.^12^

To our knowledge, this is the first report on the long-term cognitive and neuropsychological outcomes of children born to mothers with either metformin or insulin treatment of GDM. To date, in addition to previous studies of this cohort,^21,22^ only one other randomized controlled trial of metformin or insulin treatment of GDM has reported data of cognitive development of the offspring.^23^ Ijäs et al.^21^ found that motor, social, or linguistic development did not differ between 18-month-old children whose mothers were randomized to either metformin or insulin treatment of GDM. They used Finnish maternal and child welfare clinics program, in which the stage of the child's development is evaluated by a general practitioner and/or a nurse who has been specially trained in child health care, achieving high participation percentage (96%; 93 metformin/97 insulin). Tertti et al.^22^ assessed the neurodevelopment of one hundred forty-six 2-year-old offspring (68% of the original cohort) of metformin-treated or insulin-treated mothers with the Bayley Scales of Infant and Toddler Development and the Hammersmith Infant Neurological Examination, and they found no differences between the 2 medication groups. In the study by Wouldes et al.,^23^ neurodevelopment of two hundred eleven 2-year-old offspring of mothers randomized to metformin or insulin treatment of GDM was examined with the Bayley Scales of Infant Development consisting of mental development index, psychomotor development index, and behavior rating scale. Only 37% of the original cohort was studied. They reported results separately for participants from New Zealand and Australia because the results differed significantly between these countries. Neurodevelopmental outcomes were similar between treatment groups in both countries. Furthermore, in a population-based cohort study in New Zealand in patients with GDM treated with metformin or insulin, the Strengths and Difficulties Questionnaire was used to assess the difference in behavioral development before entering school between the offspring (n = 3928) of the 2 medication groups.^24^ In that study, the proportion of children having concerning scores in the Strengths and Difficulties Questionnaire and prosocial behavior scores were similar in parent and teacher ratings between the 2 groups. However, owing to the wide confidence intervals, potentially increased risk association with one or other medication could not be completely excluded.^24^

Based on previous results, it seems that fetal exposure of metformin does not affect the motor, social, behavioral, linguistic, and cognitive development of children compared with insulin treatment, when examining children before school age. Mild cognitive and neuropsychological difficulties might become evident within age and increasing demands during school years. However, in this study, neurocognitive outcomes were similar in 9-year-old children between the metformin and insulin groups. The results of this study are in line with these studies and support the hypothesis of the safety of the antenatal metformin treatment of GDM for a child's development.

It is important to observe that when metformin is used to treat polycystic ovary syndrome (PCOS), it is started during first trimester and may be continued throughout the pregnancy. Thus, possible long-term effects of metformin on offspring cognitive development might be found more likely in studies of pregnancies with PCOS mothers. As opposed to PCOS pregnancies, medication in GDM is usually started during the second or third trimester. However, in a Norwegian follow-up study of mothers with PCOS randomized to metformin or placebo treatment from the first trimester, the mean FIQ was similar in offspring aged 5 to 14 years (n = 93) in the metformin and placebo groups.^36^ It is of note that the participation rate in that study was 32%.

A major strength of this study is that the 9-year-old offspring well represent the original cohort, which allows valid comparisons between the treatment groups. Moreover, the baseline data were similar between the 9-year-old study participants and the group of nonparticipants and between the 2 study sites. Currently, this follow-up cohort of one hundred fifty-nine 9-year-old offspring whose mothers received either metformin or insulin treatment of GDM is the largest published cohort comparing long-term effects of the medication on the cognitive and neuropsychological profiles of offspring. We consider the participation rate of 51% to be satisfactory, taking into account the long period of 9 years between birth and follow-up. In addition, neuropsychological assessment covered clinically essential functions of 9-year-old children and reflect the overall picture of their neurocognitive outcome. There were some limitations in this study. First, the neuropsychological assessments were performed by 5 psychologists or final-stage psychology students, although no differences in test score medians between the psychologists were found. Second, narrative memory test (NEPSY II) results were lower than average in both medication groups. This might be related to the ceiling effect further compounded by the exhaustion from prolonged examinations. Third, the children were examined at the age of 9 years, which led to a situation in which the studied children were in different grade levels, which might have affected the level of received educational support. Fourth, the suboptimal follow-up rate may have led to some potential differences not being detected between the treatment groups, and based on post hoc power analysis regarding FSIQ, the size of this follow-up cohort does not have power to find small differences between the groups. However, a large (10-point) difference in FSIQ could be excluded on the basis of our noninferiority power calculation.

In conclusion, in this follow-up study of 9-year-old offspring of mothers with GDM randomized to metformin or insulin treatment during pregnancy, we found that metformin did not adversely affect either offspring's neurocognitive outcomes assessed by standardized tests or executive function assessed by teachers and parents. These results could obtain further confirmation by performing a follow-up study of this cohort at the end of the basic education at the age of 16 years.

## Supplementary Material
