# Supplementary material for: Metformin Versus Insulin for Gestational Diabetes: Cognitive and Neuropsychological Profiles of Children Aged 9 years
Source: J Dev Behav Pediatr. 2023 Nov 28;44(9):e642–50. doi: 10.1097/DBP.0000000000001233 (PMC10686276; doi:10.1097/DBP.0000000000001233)
Supplement: Supplementary file 1 [file jdbp-44-e642-s001.docx]

**Supplementary material**

Supplemental Table S1. Baseline maternal and neonatal characteristics of the participants of the 9-year follow-up study and the group of non-participants in the study of GDM treatment (*n* = 311).

|  | **Participants n=172** | **Non-participants n=139** | **p-value** |
| --- | --- | --- | --- |
| Insulin / Metformin | 90(52.3) / 82(47.7) | 67(48.2) / 72(51.8) | 0.47 |
| Supplemental insulin in metformin group | 21 (12.2) | 13 (9.4) | 0.41 |
| **Maternal** |  |  |  |
| Age at randomization (years) | 33.0 (29.0-36.0) | 32.0 (27.0-35.0) | 0.06ǂ |
| BMI (kg/m²), 1st antenatal visit | 28.8 (25.0-33.0) | 29.0 (26.0-33.0) | 0.13ǂ |
| Normal weight (BMI <25.0 kg/m²) | 31 (18.0) | 20 (14.4) | 0.53 |
| Overweight (BMI 25.0-29.9 kg/m²) | 68 (39.5) | 52 (37.4) | 0.37 |
| Obese (BMI≥30.0 kg/m²) | 73 (42.4) | 67 (48.2) |  |
| Total weight gain during pregnancy (kg) | 8.51±4.94 | 7.98±5.24 | 0.37 |
| Gestational weeks at randomization | 30.6 (29.3-31.9) [16.7, 34.3] | 30.4 (28.9-32.1) [19.0, 34.0] | 0.41ǂ |
| Smoking in pregnancy | 21 (12.4) | 24 (17.4) | 0.21 |
| 75g OGTT results at enrollment |  |  |  |
| fasting plasma glucose (mmol/L) | 5.4 (5.1-5.7) | 5.5 (5.2-5.9) | 0.12ǂ |
| 2-h plasma glucose (mmol/L) | 8.1 (7.0-9.4) | 7.8 (6.5-9.2) | 0.28ǂ |
| HbA1c prior randomization (%) | 5.6 (5.3-5.8) | 5.6 (5.4-5.9) | 0.13ǂ |
| HbA1c at 36 gestational weeks (%) | 5.7 (5.4-5.9) | 5.7 (5.5-6.0) | 0.11ǂ |
| Duration of medication (weeks) | 8.5 (7.0-10.6) [3.9, 22.2] | 8.7 (7.0-10.9) [2.0, 19.6] | 0.41ǂ |
| Gestational weeks at birth | 39.1 (38.4-40.1) | 39.1 (38.1-40.1) | 0.56ǂ |
| Prematurity (delivery <37 gestational wks) | 10 (5.8) | 5 (3.6) | 0.36 |
| Cesarean delivery | 37 (21.5) | 25 (18.0) | 0.44 |
| **Neonatal** |  |  |  |
| Boy/Girl (n) | 85/87 | 68/71 | 0.93 |
| Birth weight (g) | 3608.0 (3305.0-3930.0) | 3670.0 (3390.0-3930.0) | 0.51ǂ |
| Birth weight (SD) | 0.12±1.12 | 0.25±1.18 | 0.32 |
| Birth weight < -2 SD | 4 (2.3) | 4 (2.9) | 0.98 |
| Birth weight > 2 SD | 9 (5.2) | 7 (5.0) |  |
| Crown-heel length (cm) | 50.8±2.20 | 51.0±1.98 | 0.45 |
| Crown-heel length (SD) | 0.43±1.12 | 0.57±1.03 | 0.26 |
| Ponderal index (g/cm³) | 2.73±0.27 | 2.73±0.26 | 1.00 |
| Apgar score at 1 minutes | 9.0 (9.0-9.0) | 9.0 (8.0-9.0) | 0.89ǂ |
| Apgar score at 5 minutes | 9.0 (9.0-9.0) | 9.0 (9.0-9.0) | 0.83ǂ |
| Apgar score at 15 minutes | 9.0 (9.0-10.0) | 9.0 (9.0-9.0) | 0.41ǂ |
| Umbilical artery pH | 7.28 (7.22-7.33) | 7.27 (7.22-7.33) | 0.69ǂ |
| Hypoglycemia* | 35 (20.3) | 29 (20.9) | 0.91 |

Data are expressed as n (%), [min, max], mean ±SD or median (IQR). T-test or Mann-Whitney U-test (ǂ) was used for continuous and Chi-square or Fisher’s exact test for categorical variables. Abbreviations: BMI, body mass index, OGTT, oral glucose tolerance test; Ponderal index = birth weight (g) x100/crown-heel length (cm)³. *Need for intravenous glucose.

Supplemental Table S2. Baseline maternal and neonatal characteristics of the participants of the 9-year follow-up study (*n* = 172). Comparison between groups treated with metformin or insulin for GDM.

|  | **Metformin n=82** | **Insulin**  **n=90** | **p-value** |
| --- | --- | --- | --- |
| **Maternal** |  |  |  |
| Age at randomization (years) | 33.5 (29.0-36.0) | 33.0 (29.0-36.0) | 0.80ǂ |
| BMI (kg/m²), 1st antenatal visit | 29.0 (25.0-33.0) | 28.0 (25.5-33.0) | 0.68ǂ |
| Normal weight (BMI <25.0 kg/m²) | 18 (22.0) | 13 (14.4) | 0.12 |
| Overweight (BMI 25.0-29.9 kg/m²) | 26 (31.7) | 42 (46.7) |  |
| Obese (BMI≥30.0 kg/m²) | 38 (46.3) | 35 (38.9) |  |
| Total weight gain (kg) during pregnancy | 8.4±4.62 | 8.6±5.24 | 0.85 |
| Gestational weeks at randomization | 30.4 (29.4-31.9) [16.7, 34.0] | 30.6 (29.3-32.0) [20.3, 34.0] | 0.66ǂ |
| Smoking in pregnancy | 6 (7.4) | 15 (16.9) | 0.06 |
| 75g OGTT results at enrollment |  |  |  |
| fasting plasma glucose (mmol/L) | 5.4 (5.0-5.7) | 5.4 (5.2-5.8) | 0.28ǂ |
| 2-h plasma glucose (mmol/L) | 8.4±1.93 | 8.1±1.79 | 0.36 |
| HbA1c prior randomization (%) | 5.6±0.42 | 5.6±0.38 | 0.59 |
| HbA1c at 36 gestational weeks (%) | 5.6±0.34 | 5.7±0.38 | 0.56 |
| Duration of medication (weeks) | 8.6 (7.0-10.6) [4.3, 22.2] | 8.4 (6.8-10.6) [2.0, 18.7] | 0.69ǂ |
| Gestational weeks at birth (weeks) | 39.0 (38.4-40.1) | 39.1 (38.4-40.3) | 0.70ǂ |
| Prematurity (delivery <37 gestational wks) | 6 (7.3) | 4 (4.4) | 0.42 |
| Cesarean delivery | 18 (22.0) | 19 (21.1) | 0.89 |
| **Neonatal** |  |  |  |
| Boy/Girl (n) | 42/40 | 43/47 | 0.65 |
| Birth weight (g) | 3610.6±488.8 | 3575.8±519.2 | 0.65 |
| Birth weight (SD) | 0.15±1.08 | 0.09±1.16 | 0.71 |
| Birth weight < -2 SD | 3 (3.7) | 1 (1.1) | 0.41 |
| Birth weight > 2 SD | 5 (6.1) | 4 (4.4) |  |
| Crown-heel length (cm) | 51.0 (50.0-52.0) | 51.0 (49.5-52.0) | 0.54ǂ |
| Crown-heel length (SD) | 0.45 (-0.2-1.2) | 0.40 (-0.2-1.2) | 0.77ǂ |
| Ponderal index (g/cm³) | 2.73±0.26 | 2.73 ±0.28 | 0.94 |
| Apgar score at 1minutes | 9.0 (9.0-9.0) | 9.0 (8.0-9.0) | 0.22ǂ |
| Apgar score at 5minutes | 9.0 (9.0-9.0) | 9.0 (9.0-9.0) | 0.87ǂ |
| Apgar score at 15 minutes | 9.0 (9.0-10.0) | 9.0 (9.0-10.0) | 0.56ǂ |
| Umbilical artery pH | 7.27±0.09 | 7.27±0.08 | 0.92 |
| Hypoglycemia* | 18 (22.0) | 17 (18.9) | 0.62 |

Data are expressed as n (%), [min, max], mean ±SD or median (IQR). T-test or Mann-Whitney U-test (ǂ) was used for continuous and Chi-square or Fisher’s exact test for categorical variables. Abbreviations: BMI, body mass index; OGTT, oral glucose tolerance test; Ponderal index = birth weight (g) x100/crown-heel length (cm)³. *Need for intravenous glucose.

Supplemental Table 3. Proportion of children whose results were below average; in cognitive profile by WISC-IV indexes (below 85 standard points), in neuropsychological measures by NEPSY II (below 8 standard points), in reading fluency by LUKILASSE (below -1.34 sd) and in school related information at 9 years of age. Psychological assessments were performed in 159 participants. Comparison between offspring of the mothers treated with metformin or insulin for GDM.

|  | All Children | | | Boys | | | Girls | | |
| --- | --- | --- | --- | --- | --- | --- | --- | --- | --- |
|  | Metformin | Insulin | p-value | Metformin | Insulin | p-value | Metformin | Insulin | p-value |
| **Cognitive development**;  WISC-IV | n=77 | n= 82 |  | n= 39 | n= 38 |  | n=38 | n=44 |  |
| FSIQ < -1SD (<85 std points), n (%) | 22 (28.6) | 13 (16.5) | 0.070 | 13 (33.3) | 8 (21.6) | 0.25 | 9 (23.7) | 5 (11.9) | 0.17 |
| VCI < -1SD (<85 std points), n (%) | 12 (15.6) | 9 (11.4) | 0.44 | 6 (15.4) | 6 (16.2) | 0.92 | 6 (15.8) | 3 (7.1) | 0.23† |
| PRI < -1SD (<85 std points), n (%) | 21 (27.3) | 15 (18.3) | 0.18 | 11 (28.2) | 8 (21.1) | 0.47 | 10 (26.3) | 7 (15.9) | 0.25 |
| WMI < -1SD (<85 std points), n (%) | 13 (16.9) | 15 (18.3) | 0.82 | 8 (20.5) | 5 (13.2) | 0.39 | 5 (13.2) | 10 (22.7) | 0.26 |
| PSI < -1SD (<85 std points), n (%) | 10 (13.0) | 11 (13.4) | 0.94 | 6 (15.4) | 7 (18.4) | 0.72 | 4 (10.5) | 4 (9.1) | 0.83† |
| **Neuropsychological measures;**  NEPSY II | n=73 | n=78 |  | n=37 | n=35 |  | n=36 | n=43 |  |
| Comprehension of Instructions, <8, n (%) | 16 (21.9) | 12 (15.4) | 0.30 | 10 (27.0) | 5 (14.3) | 0.18 | 6 (16.7) | 7 (16.3) | 0.96 |
| Narrative memory, <8, n (%) | 41 (56.2) | 39 (50.6) | 0.50 | 23 (62.2) | 22 (62.9) | 0.95 | 18 (50.0) | 17 (40.5) | 0.40 |
| **Academic measures**;  LUKILASSE | n=74 | n=78 |  | n=37 | n=37 |  | n=37 | n=41 |  |
| Clearly below grade level (<-1.34) | 11 (14.9) | 14 (17.9) | 0.61 | 9 (24.3) | 7 (18.9) | 0.57 | 2 (5.4) | 7 (17.1) | 0.11 |

Data are expressed as n (%). Chi-square or Fisher’s exact test (†) was used. Abbreviations: FSIQ, Full-scale intelligence quotient; LUKILASSE, Screening test for reading, writing and calculus for 1st to 6th grades; NEPSY-II, Developmental Neuropsychological Assessment; PRI, Perceptual Reasoning Index; PSI, Processing Speed Index; TMT, Trail Making Test; VCI, Verbal Comprehension Index; WISC-IV, Wechsler Intelligence scale for Children, Fourth Edition.

Supplemental Table 4. Executive functioning in daily life at 9 years of age as assessed by the teacher and parent. 4.a Medians of BRIEF subscales and 4.b. proportion of 9-year-old children who had clinically significant problems (scores above 64) in executive functions at school and at home by BRIEF. Comparison between offspring of the mothers treated with metformin or insulin for GDM.

| 4.a. | All children | | |
| --- | --- | --- | --- |
|  | Metformin | Insulin | P value |
| **BRIEF Profile at School** | n=78 | n=84 |  |
| Inhibit | 49.0 (45-60) | 46.0 (45-55) | 0.16 |
| Shift | 47.0 (45-57) | 47.0 (45-55) | 0.98 |
| Emotional control | 47.0 (46-61.5) | 48.0 (46-54) | 0.89 |
| Initiate | 50.5 (43-60) | 51.0 (45-62) | 0.30 |
| Working memory | 50.5 (44-65) | 51.0 (44-64) | 0.89 |
| Plan/organize | 47.0 (43-55) | 49.0 (43-55) | 0.69 |
| Organization of materials | 46.0 (46-54) | 48.0 (46-57) | 0.17 |
| Monitor | 52.5 (45-63) | 49.5 (45-57) | 0.42 |
| **BRIEF Profile at Home** | n=81 | n=89 |  |
| Inhibit | 44.0 (40-50) | 42.0 (40-50) | 0.39 |
| Shift | 43.0 (39-47) | 41.0 (39-47) | 0.17 |
| Emotional control | 45.0 (38-54) | 45.0 (38-54) | 0.86 |
| Initiate | 50.0 (44-59) | 50.0 (46-59) | 0.79 |
| Working memory | 45.0 (39-54) | 44.0 (39-49) | 0.20 |
| Plan/organize | 39.0 (37-45) | 39.0 (38-45) | 0.87 |
| Organization of materials | 45.0 (42-57) | 48.0 (42-54) | 0.88 |
| Monitor | 44.0 (38-52) | 41.0 (37-49) | 0.089 |
|  |  |  |  |
| 4.b. | All Children | | |
|  | Metformin | Insulin | P value |
| **BRIEF Clinically significant problems at school** | n=81 | n=89 |  |
| Inhibit | 16 (21) | 11 (13) | 0.21 |
| Shift | 10 (13) | 12 (14) | 0.79 |
| Emotional control | 17 (22) | 11 (13) | 0.14 |
| Initiate | 13 (17) | 17 (20) | 0.56 |
| Working memory | 20 (26) | 19 (23) | 0.65 |
| Plan/organize | 8 (10) | 8 (10) | 0.88 |
| Organization of materials | 8 (10) | 16 (19) | 0.12 |
| Monitor | 16 (21) | 12 (14) | 0.30 |
| **BRIEF Clinically significant problems at home** | n=78 | n=84 |  |
| Inhibit | 3 (4) | 5 (6) | 0.56† |
| Shift | 2 (3) | 1 (1) | 0.51† |
| Emotional control | 5 (6) | 3 (3) | 0.39† |
| Initiate | 11 (14) | 12 (14) | 0.99 |
| Working memory | 4 (5) | 3 (3) | 0.61† |
| Plan/organize | 1 (1) | 1 (1) | 0.95† |
| Organization of materials | 4 (5) | 3 (3) | 0.61† |
| Monitor | 3 (4) | 2 (2) | 0.58† |

Data are expressed as median (IQR) or n (%). Scores above 64 are used to indicate clinically significant problems. Mann-Whitney U-test was used for continuous and Chi-square or Fisher’s exact test (†) for categorical variables Abbreviations: BRIEF, The Behavior Rating Inventory of Executive Function.
